# Supplementary material for: An Overview of Aquatica Fu et al., a Phylogeny of Aquatic Fireflies Using Mitochondrial Genomes, a Description of Two New Species, and a New Record of Aquatic Fireflies in China (Coleoptera: Lampyridae: Luciolinae)
Source: Insects. 2024 Jan 3;15(1):31. doi: 10.3390/insects15010031 (PMC10817006; doi:10.3390/insects15010031)
Supplement: Supplementary file 1 [file insects-15-00031-s001.zip › insects-2708040-supplementary.pdf]

insects

Supplementary Materials

An Overview of *Aquatica* Fu et al., a Phylogeny of Aquatic Fireflies Using Mitochondrial Genomes, a Description of Two New Species, and a New Record of Aquatic Fireflies in China (Coleoptera: Lampyridae: Luciolinae)

Xinhua Fu [1,2,3] and Lesley Ballantyne [4,*]

[1] College of Plant Science and Technology, Huazhong Agricultural University, Wuhan 430070, China; fireflyfxh@mail.hzau.edu.cn
[2] Hubei Insect Resources Utilization and Sustainable Pest Management Key Laboratory, Wuhan 430070, China
[3] Firefly Conservation Research Centre, 1 Shizishan Street, Wuhan 430070, China
[4] School of Agricultural, Environmental and Veterinary Sciences, Charles Sturt University, P.O. Box 588, Wagga Wagga 2678, Australia
* Correspondence: lballantyne@csu.edu.au

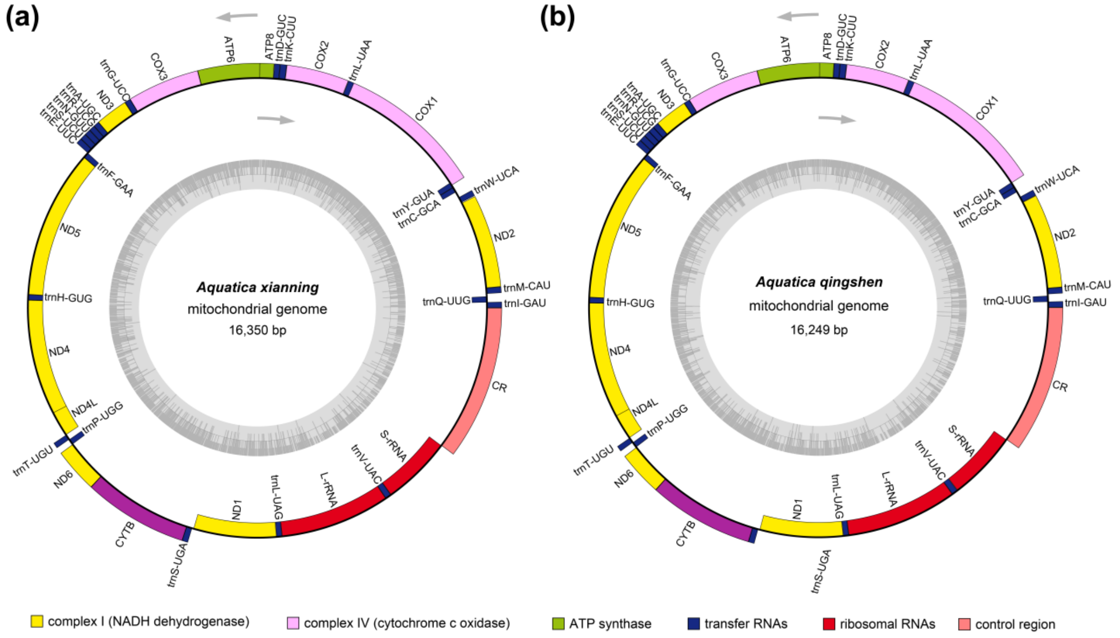

Figure S1. Circular maps of 2 newly sequenced mitogenomes of *Aquatica xianning* sp. nov. and *Aquatica qingshen* sp nov. Genes are represented by different colored blocks, as presented in the legend below the maps. Colored blocks outside of each ring indicate that the genes were on the direct strand, while colored blocks in the rings indicate that the genes were located on the reverse strand.

Table S1. *Aquatica* species and distribution.

Species	China Mainland	Taiwan/ Formosa	Japan	Location of types	Larvae associated	Females associated	References for pictures of species
A. ficta (Olivier, 1909)	*	*		MNHN	√	√	[1,4,13]
A. hydrophila (Jeng *et al*., 2003)		*		NMNS	√	√	[1,16]
A. lateralis (Motschulsky, 1860)	*		*	ZMUM	√	√	[1,4,13]
A. leii (Fu *et al.* 2006)	*			NHMHAU	√	√	[1,13]
A. qingshen sp. nov.	*			NHMHAU	√	√	This study
A. wuhana (Fu *et al.* 2010)	*			NHMHAU	√	√	[1,13]
A. xianning sp. nov.	*			NHMMAU	√	√	This study

* Distribution; √ confirmation

Table S2. *Aquatica* females and characteristics.

Species	1	2	3	4	5	6	7
A. leii (Fu *et al*. 2006)	E	H	In cop	strips	√	√	√
A. ficta (Olivier, 1909)	E	H	In cop	strips	√	√	√
A. hydrophila (Jeng *et al*., 2003)	E	H	In cop	NI	NI	NI	NI
A. lateralis (Motschulsky, 1860)	E	H	In cop	strips	√		
A. qingshen sp. nov.	E	H	In cop	strips	√		√
A. wuhana (Fu *et al*. 2010)	E	H	In cop	strips	√	√	√
A. xianning sp. nov.	E	H	In cop.	strips	√		√

* 1. Elytra. E = fully developed. 2. Hind wings. H = fully developed. 3. Evidence for association. 4. Bursa plates. 5. Median oviduct plate. 6. Spermatophore observed. 7. FAG observed. NI = not investigated.

Table S3. Matrix p-distance of 20 species of fireflies.

Species	*Ab.anceyi*	*Ab. cerata*	*Ab.terminalis*	*Aq.ficta*	*Aq.lateralis japanese*	*Aq.lateralis chinese*	*Aq. leii*	*Aq. xianning*	*Aq. qingshen*	*Aq.wuhana*	*As. circumdata*	*C. bilineatus*	*C. costipennis*	*C. fulvocapitalis*	*L. curtithorax*	*N. cruciata*	*Pt.maipo*	*Pyg.qingyu*	*Pyr.rufa*	*S. substriata*
Ab.anceyi	0.0000																			
Ab. cerata	0.1940	0.0000																		
Ab.terminalis	0.1591	0.1133	0.0000																	
Aq.ficta	0.2660	0.1718	0.1778	0.0000																
Aq.lateralis japanese	0.2803	0.1828	0.1897	0.1280	0.0000															
Aq.lateralis chinese	0.2679	0.1769	0.1807	0.1300	**0.0945**	0.0000														
Aq. leii	0.2675	0.1729	0.1786	**0.0036**	0.1290	0.1303	0.0000													
Aq. xianning	0.2846	0.1917	0.1954	0.1415	0.1517	0.1517	0.1420	0.0000												
Aq. qingshen	0.2714	0.1815	0.1857	0.1267	0.1415	0.1418	0.1271	0.1084	0.0000											
Aq.wuhana	0.2622	0.1707	0.1791	0.1211	0.1430	0.1421	0.1215	0.1544	0.1395	0.0000										
As. circumdata	0.2925	0.2022	0.2084	0.2170	0.2270	0.2219	0.2181	0.2319	0.2177	0.2211	0.0000									
C. bilineatus	0.3021	0.2071	0.2155	0.2238	0.2351	0.2314	0.2248	0.2361	0.2298	0.2244	0.2406	0.0000								
C. costipennis	0.3024	0.2015	0.2128	0.2238	0.2343	0.2300	0.2251	0.2400	0.2264	0.2212	0.2357	0.0814	0.0000							
C. fulvocapitalis	0.2953	0.2120	0.2202	0.2292	0.2405	0.2360	0.2295	0.2481	0.2378	0.2274	0.2481	0.1632	0.1650	0.0000						
L. curtithorax	0.2507	0.1576	0.1629	0.1740	0.1858	0.1720	0.1751	0.1963	0.1803	0.1792	0.2069	0.2113	0.2119	0.2197	0.0000					
N. cruciata	0.2800	0.1838	0.1883	0.1509	0.1589	0.1629	0.1520	0.1612	0.1513	0.1626	0.2222	0.2352	0.2336	0.2462	0.1825	0.0000				
Pt.maipo	0.2641	0.1701	0.1772	0.1849	0.2006	0.1938	0.1857	0.2039	0.1941	0.1872	0.2098	0.1984	0.1949	0.2022	0.1780	0.1974	0.0000			
Pyg.qingyu	0.2151	0.1258	0.1333	0.1638	0.1804	0.1685	0.1642	0.1832	0.1678	0.1665	0.1947	0.2030	0.2001	0.2096	0.1484	0.1757	0.1682	0.0000		
Pyr.rufa	0.3839	0.2981	0.2984	0.3137	0.3248	0.3147	0.3136	0.3253	0.3147	0.3173	0.3138	0.3130	0.3142	0.3217	0.2980	0.3193	0.2984	0.2883	0.0000	
S. substriata	0.2659	0.1733	0.1754	0.1827	0.1898	0.1861	0.1827	0.1989	0.1895	0.1876	0.2144	0.2202	0.2163	0.2259	0.1751	0.1929	0.1855	0.1649	0.3097	0.0000

P-distances mentioned in main text are marked in different colours. Red in yellow shade shows genetic distance between *Aq. leii* and *Aq.ficta*; Blue in yellow shade shows genetic distance between *Aq.lateralis* Chinese and *Aq.lateralis* Japanese; green shows genetic distance between *Aq. xianning* and other firefly species; brown shows genetic distance between *Aq. qingshen* and other firefly species.
